# Molecular and biochemical characterization of calmodulin from *Echinococcus granulosus*

**DOI:** 10.1186/s13071-017-2545-2

**Published:** 2017-12-04

**Authors:** Ning Wang, Xiuqin Zhong, Xingju Song, Xiaobin Gu, Weiming Lai, Yue Xie, Xuerong Peng, Guangyou Yang

**Affiliations:** 1 Department of Parasitology, College of Veterinary Medicine, Sichuan Agricultural University, Chengdu, 611130 China; 2 Department of Chemistry, College of Life and Basic Science, Sichuan Agricultural University, Chengdu, 611130 China

**Keywords:** *Echinococcus granulosus*, Calmodulin, Ca^2+^-binding protein, Immunohistochemical localization, Quantitative real-time PCR

## Abstract

**Background:**

*Echinococcus granulosus* is a harmful cestode parasite that causes cystic echinococcosis in humans as well as various livestock species and wild animals. Calmodulin (CaM), a Ca^2+^ sensor protein, is widely expressed in eukaryotes and mediates a variety of cellular signaling activities.

**Methods:**

In the present study, the cDNA encoding CaM in *Echinococcus granulosus* (rEgCaM) was successfully cloned and the molecular and biochemical characterizations carried out. The antigenicity and immunoreactivity of rEgCaM was detected and the preliminary enzyme-linked immunosorbent assay (ELISA)-based serodiagnostic potential of EgCaM was assessed. The locations of this protein in the adult worm and larval stage, and the mRNA expression in different states of *E. granulosus* protoscoleces (PSCs) were defined clearly. Moreover, the Ca^2+^-binding properties of EgCaM were measured.

**Results:**

rEgCaM is a highly conserved calcium-binding protein, consisting of 149 amino acids. Immunoblotting analysis revealed that rEgCaM could be identified using *E. granulosus* infected sheep serum. The use of rEgCaM as an antigen was evaluated by indirect ELISA which exhibited a high sensitivity (90.3%), but low specificity (47.1%). rEgCaM was ubiquitously expressed in protoscoleces and adults of *E. granulosus*, as well as in the germinal layer of the cyst wall. The mRNA expression level of rEgCaM was increased from the start of H_2_O_2_ exposure and then gradually decreased because of the increased apoptosis of PSCs. In electrophoretic mobility tests and 1-anilinonaphthalene-8-sulfonic acid assays, rEgCaM showed a typical characteristic of a calcium-binding protein.

**Conclusions:**

To our knowledge, this is the first report on CaM from *E. granulosus* and rEgCaM is likely to be involved in some important biological function of *E. granulosus* as a calcium-binding protein.

## Background

Cystic echinococcosis (CE), also called hydatid disease, is a serious zoonotic parasitic disease caused by the larval form of *Echinococcus granulosus* and is an important public health issue in both developed and developing countries [1, 2]. The larval stage of *E. granulosus* infects livestock and humans, while the adult worm parasitizes the small intestine of canids. The metacestode larva is a unilocular, fluid-filled cyst which includes a germinal layer, a laminated layer, and an external layer derived from dead host cells and fibrosis. CE results in an estimated 1–3 million disability-adjusted life years (DALYs) globally per annum and 20–90% of CE prevalence is observed in domestic animals, leading to an annual economic loss of approximately US$3 billion [3]. The World Health Organization included echinococcosis as one of the 17 neglected tropical diseases in its strategic plan from 2008 to 2015 [4].

Calmodulin (CaM), a small calcium sensor protein, is one of the most evolutionarily ancient proteins in eukaryotes [5]. The functions of CaM include Ca^2+^ binding and conversion of Ca^2+^ signals though downstream proteins to regulate various physiological processes, such as muscle contraction, metabolism and cell motility [6, 7]. The structure of CaM is characterized by two globular heads joined by an extended α-helical linker; each of the globular heads includes two EF-hands domains that can bind to a calcium ion (Ca^2+^) [8, 9]. After binding to Ca^2+^, CaM changes its conformation, exposing more hydrophobic residues in order to interact with diverse target proteins [10, 11]. In *Caenorhabditis elegans*, 56 Ca^2+^-bound-calmodulin binding proteins were identified using mRNA-display, including heat shock proteins, myosin family members, CaM-dependent kinases, protein phosphatases and phosphodiesterases [12].

Although CaM has been widely studied and well-characterized in many organisms, it has not been cloned or characterized in *Echinococcus* spp.. Only in *Echinococcus multilocularis* (a species that has a close genetic relationship with *E. granulosus*), was CaM predicted as a potential drug target with a high score and available chemical leads (known drugs or approved compounds) [13]. In the present study, *E. granulosus* calmodulin (EgCaM) was identified and characterized. The antigenicity and immunoreactivity of rEgCaM were detected and the preliminary enzyme-linked immunosorbent assay (ELISA)-based serodiagnostic potential of EgCaM was assessed. The locations of this protein in the adult worm and larval stage, and its mRNA expression in different states of protoscoleces (PSCs) were clearly defined. Moreover, the Ca^2+^-binding properties of EgCaM were assessed.

## Methods

### Animals and parasites

*Echinococcus granulosus* protoscoleces (PSCs) and cyst walls were isolated aseptically using a previously reported method from liver hydatid cysts of naturally infected cattle presented for routine slaughter in an abattoir in Qinghai Province, China [14]. Two thousand PSCs were cultured in 1 ml of Roswell Park Memorial Institute (RPMI) 1640 medium with 10% bovine serum albumin (BSA; Hyclone, Logan, USA), 100 U/ml penicillin, and 100 μg/ml streptomycin (Sigma-Aldrich, St. Louis, USA). Adult worms were obtained from a 5-month-old dog after 35 days after artificial infection with PSCs. Four New Zealand white rabbits were prepared to produce polyclonal antibodies.

### Sera

Sera from 31 sheep naturally infected with *E. granulosus* were isolated, and 24 negative sera were isolated from healthy sheep with no cysts at autopsy. All samples were collected from the slaughterhouse in Xinjiang Province. Sera (seven samples) from sheep naturally infected with *Taenia multiceps* and sera (10 samples) from goats naturally infected with *Cysticercus tenuicollis* were collected from the slaughterhouse in Sichuan Province.

### Bioinformatic analysis

The open reading frame (ORF) was identified using an ORF finder tool and analyzed using BLASTp at NCBI. Proteomics tools on the ExPaSy website (http://www.expasy.org/) were used to analyze the physicochemical parameters, signal peptide and transmembrane regions of the amino acid sequence. A phylogenetic tree (neighbor-joining tree) for calmodulin was constructed using MEGA v. 5.

### Cloning, expression and purification of recombinant EgCaM (rEgCaM)

Total parasite RNA from PSCs was extracted using a commercial kit (Cowin Biotech, Beijing, China) following the manufacturer’s instructions. cDNA was synthesized using 1 μg of total RNA as the template for the Reverse Transcription System (Thermo Fisher, Waltham, USA). The sequence encoding calmodulin was amplified by polymerase chain reaction (PCR) using the following specific primers: sense primer 5′-CGG GAT CCA TGG CTG ACC AAC TTA CA-3′ and antisense primer 5′-CCC TCG AGC TAC TTC GAC TGC ATC ATC-3′, which introduce *BamH*I and *Xho*I restriction enzyme sites (underlined), respectively. The PCR protocol included 30 cycles of 95 °C for 30 s, 62 °C for 30 s, and 72 °C for 1 min. After purification, the PCR product was ligated into the T&A cloning vector and transformed into *Escherichia coli* DH5α. Plasmid DNA digested using *BamHI* and *XhoI* restriction enzymes was cloned into expression vector pET-28a (Novagen, Madison, USA) and transformed into *E. coli* BL21 (DE3) (Cowin Biotech, Beijing, China). An *E. coli* clone with the correct DNA sequence was cultured in Luria Bertani broth, and expression of rEgCaM was induced using 1 mM isopropyl-1-thio-β-D -galactopyranoside (IPTG) at 37 °C for 6 h. The rEgCaM with a poly-histidine tag was affinity purified from bacterial lysate using Ni-NTA His-tag resin (Qiagen, Hilden, Germany). The purified rEgCaM was analyzed by 15% sodium dodecyl sulfate polyacrylamide gel electrophoresis (SDS-PAGE). The final concentration of purified rEgCaM was determined using a BCA protein assay kit (Beyotime, Jiangsu, China).

### Preparation of polyclonal antibodies against rEgCaM

Four rabbits were used to produce the polyclonal antibodies. Rabbit sera were collected before immunization to provide a reagent for negative controls. For the first immunization, 200 μg of rEgCaM emulsified with an equal volume of Freund’s complete adjuvant (Sigma-Aldrich) was injected subcutaneously. The second and third injections to boost immunization were given by mixing 100 μg of protein with an equal volume of Freund’s incomplete adjuvant at 2-week intervals. Two weeks after the final injection, rabbit antisera were collected. The antibody titer was determined by enzyme-linked immuno-sorbent assay (ELISA). Immunoglobulin G (IgG) was further isolated from the antisera using a Protein G-Sepharose column (Bio-Rad, Richmond, USA).

### Western blotting

Purified rEgCaM was separated by SDS-PAGE and subsequently transferred onto a polyvinylidene difluoride membrane (Millipore, Schwalbach, Germany). The membranes were blocked with 5% (*w*/*v*) skimmed milk at room temperature for 2 h, and then incubated with *E. granulosus* positive sheep sera or anti-rEgCaM rabbit sera (1:150 *v*/v dilutions with 0.01 M PBS) overnight at 4 °C. After washing with TBST (40 mM Tris-HCl, 0.5 M NaCl, 0.5% Tween-20, pH 7.4) the membranes were incubated with horseradish peroxidase (HRP)-conjugated goat anti-rat antibody for 1 h. Immunoreactive bands were detected using diaminobenzidine (DAB) reagent (Tiangen, Beijing, China) according to the manufacturer’s instructions. Total PSCs extract was used to detect the specificity and sensitivity of rabbit anti-rEgCaM IgG, used the method described above. Non-infected sheep and pre-immunized rabbit sera were used as negative controls.

### Development of an indirect ELISA

Ninety-six-well microtiter plates were coated with 100 μl of two-fold diluted rEgCaM antigen (ranging from 1:20–1:2560) diluted in 0.1 M carbonate buffer (pH 9.6) at 4 °C overnight. After washing three times with phosphate-buffered saline-Tween 20 (PBST) to remove non-adsorbed antigen, the plates were blocked with 5% skimmed milk (*w*/*v*) in PBS at 37 °C for 1 h. Positive and negative sera isolated from sheep were two-fold diluted in PBS ranging from 1:20 to 1:640 (3.2 μg/well to 0.1 μg/well) and incubated at 37 °C for 1 h. After washing, 100 μl of rabbit anti-sheep HRP-conjugated antibody (diluted to 1:3000 with PBS) was added to each well and incubation continued at 37 °C for 1 h. After a final wash, the enzyme reaction was visualized by the addition of 3,3′,5,5′-tetramethylbenzidine (TMB) at room temperature for 15 min and stopped with Stop Solution (0.5 M phosphoric acid). The OD value (optical density) was measured at 450 nm using a microplate reader.

### Evaluation and statistical analysis

The best dilutions of rEgCaM antigen and sera were determined, and then 62 sheep sera (31 for *E. granulosus*-positive, 7 for *T. multiceps*-positive and 24 for negative control) and 10 goat sera (for *C. tenuicollis*-positive) were serodiagnosed using the indirect ELISA described as above. *Echinococcus granulosus*-positive and negative sera were used in all plates, acting as the intra-plate controls. The sensitivity of this method was assessed by the percentage value of ELISA positive and true positives, while the specificity was evaluated by the cross-reaction with *T. multiceps* and *C. tenuicollis*-positive sera.

The negative cut-off was defined as the mean value +3× standard deviations (SD) from the OD values obtained from 24 negative sera. The significance of comparisons between test sera groups was estimated by ANOVA (SPSS Inc., Chicago, IL, USA).

### Immunohistochemical localization of EgCaM

Fresh adult worms, PSCs and cyst walls of *E. granulosus* were fixed with 4% paraformaldehyde, embedded in paraffin wax, and then sliced into 5 μm thick sections. The sections were dewaxed, rehydrated, treated to inactivate endogenous peroxidase activity and incubated in 5% BSA in phosphate buffered saline (PBS) for 1 h at room temperature. Then, the sections were then incubated with anti-rEgCaM rabbit IgG or native rabbit IgG (1:500 *v/v* dilutions in PBS) overnight at 4 °C. After washing three times with PBS, the sections were then incubated with fluorescein isothiocyanate (FITC)-conjugated goat anti-rabbit IgG (1:200 *v/v* dilution in 0.1% Evan’s Blue) for 1 h at 37 °C in the dark. After washing three times with PBS, glycerine was added to the sections and images were observed under a fluorescence microscope.

### rEgCaM mRNA expression of PSCs in different states

PSCs were cultured in 12-well microplates and incubated with 5 mM H_2_O_2_ for 6 h at 37 °C to induce cell apoptosis and death of PSCs. Every hour, PSCs were collected and stored at −80 °C for further study.

Total RNA was extracted and the corresponding cDNA was obtained as described above. To quantify the transcript level of rEgCaM in different states of PSCs, quantitative real-time reverse transcription PCR were conducted [15]. The primers for rEgCaM were 5′-GAA GGA TAC CGA TAG TGA GGA AGA-3′ and 5′-ATC ATT TCG TCA ACC TCC TCG TC-3′. The primers of the housekeeping gene *ACTB* (encoding β-actin) were 5′-ATG GTT GGT ATG GGA CAA AAG G-3′ and 5′-TTC GTC ACA ATA CCG TGC TC-3′. The data were calculated using the 2^-∆∆CT^ method.

### Ca^2+^-binding properties of rEgCaM

To investigate the Ca^2+^-binding properties of rEgCaM, denaturing and native gel electrophoresis were used. rEgCaM (0.5 mM) was incubated with CaCl_2_ (50 mM) and EDTA (50 mM), respectively, on ice for 1 h. The control was untreated rEgCaM protein. Subsequently, equal volumes of SDS gel loading buffer were added and the samples were heated in boiling water for 5 min, followed by separation on 15% denaturing polyacrylamide gels. Native gel electrophoresis followed a similar procedure but without SDS, and the mixture was directly loaded onto the gels (not heated). Proteins were visualized by staining with Coomassie Blue.

### ANS fluorescence

1-anilinonaphthalene-8-sulfonic acid (ANS) is a widely used fluorescent probe. rEgCaM (0.5 mM) and ANS (3 mM; Sigma-Aldrich, USA) in PBS were incubated with 50 mM CaCl_2_ and 50 mM EDTA, respectively, on ice for 1 h. rEgCaM alone (0.5 mM) was the control. The results were measured in a Spectra Max M5 microplate fluorometer at 25 °C with 380 nm as the excitation wavelength for ANS. The emission wavelength scans were collected between 400 and 700 nm. The experiments were repeated three times.

## Results

### Sequence analysis of calmodulin

**Fig. 1:**
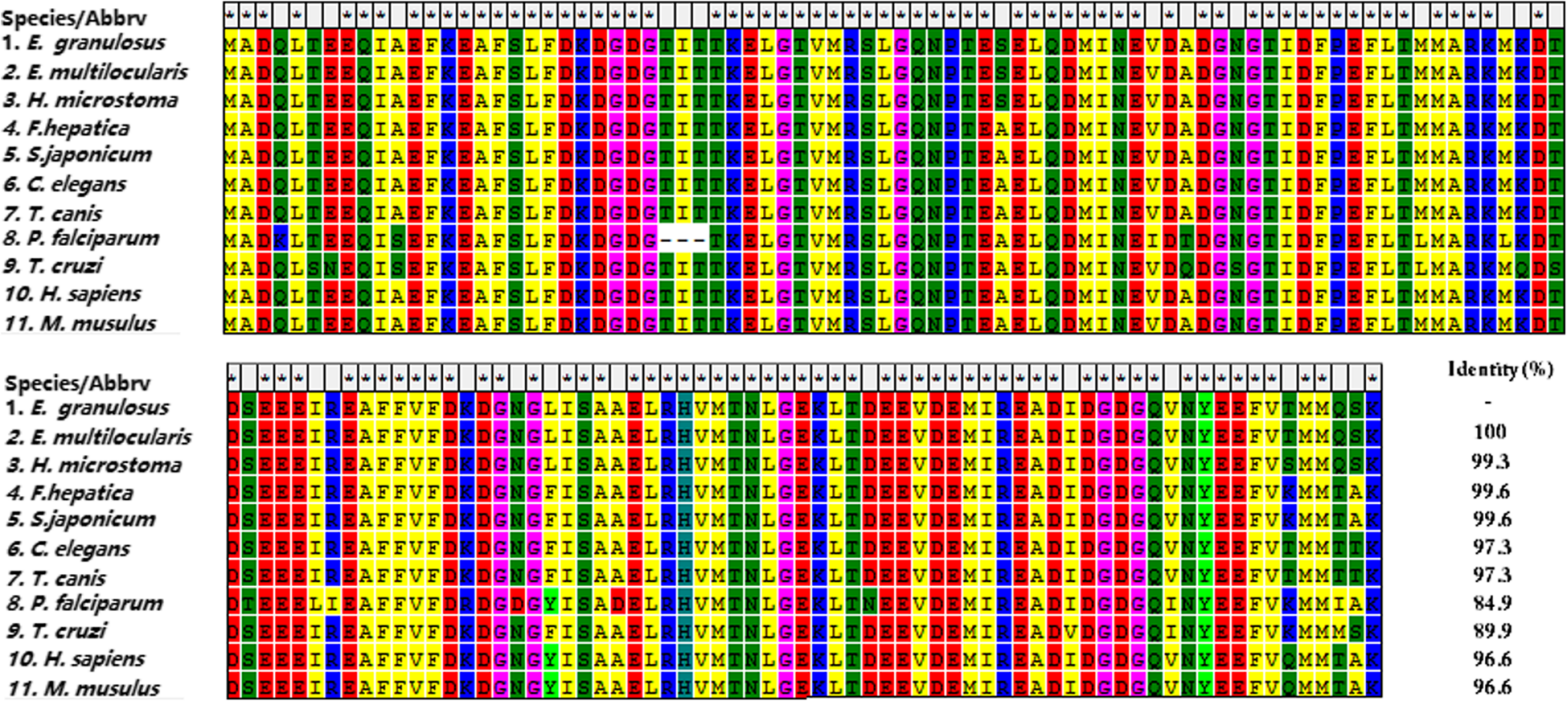
Multiple alignment of the deduced amino acid sequence of *Echinococcus granulosus* calmodulin with sequences of related proteins from other parasites and hosts. *Echinococcus multilocularis* (CDS37648.1), *Hymenolepis microstoma* (CDS28106.1), *Fasciola hepatica* (CAL91032.1), *Schistosoma japonicum* (AAW27335.1), *Caenorhabditis elegans* (NP_503386.1), *Toxocara canis* (KHN73369.1), *Plasmodium falciparum* (AAA29509.1), *Trypanosoma cruzi* (XP_808093.1), *Homo sapiens* (NP_001734.1), and *Mus musculus* (NP_033920.1)

**Fig. 2:**
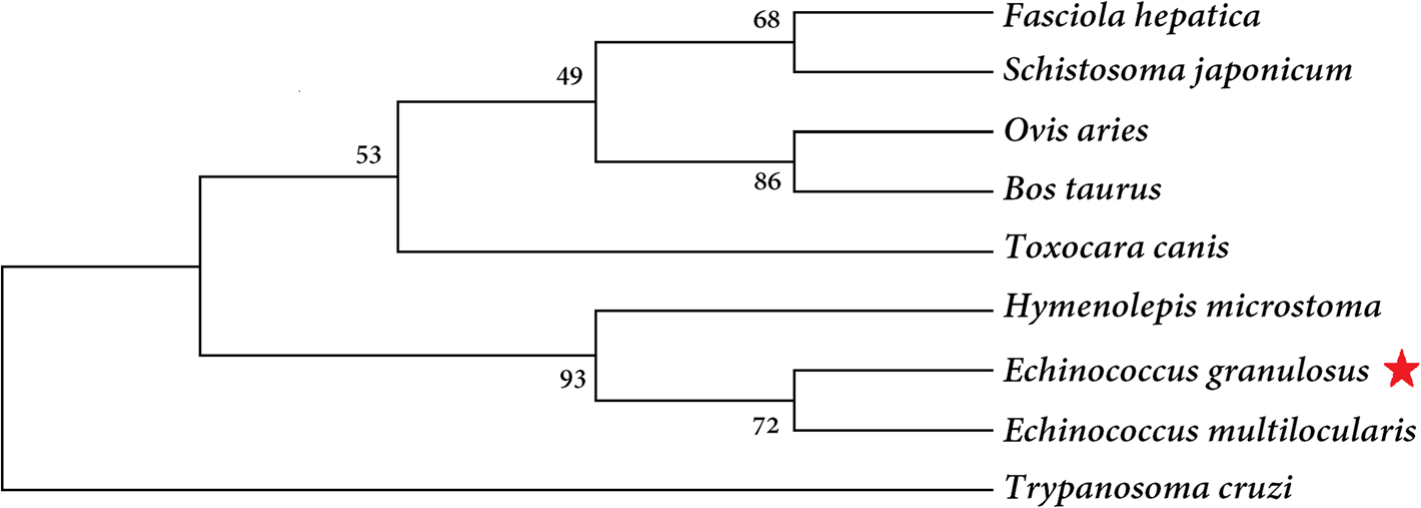
Phylogenetic tree of calmodulin. The tree was built using the neighbor-joining method in MEGA v. 5. The bootstrap values are shown at the branching points (1000 replications)

The cDNA sequence of EgCaM had an open reading frame of 450 bp (GenBank: KR153481) encoding a protein of 149 amino acids with a theoretical molecular weight of 16.8 kDa (isoelectric point, pI = 4.09). The instability index was calculated as 33.43. No signal peptide or transmembrane regions were found in the deduced amino acid sequence. BLASTp showed that the amino acid sequence of EgCaM shared 84.9–100% identity with CaMs from *E. multilocularis*, *Hymenolepis microstoma*, *Fasciola hepatica*, *Schistosoma japonicum*, *Caenorhabditis elegans*, *Toxocara canis*, *Plasmodium falciparum*, *Trypanosoma cruzi*, *Homo sapiens* and *Mus musculus* (Fig. 1). Four Ca^2+^-binding domains were located at residues 21–32, 57–68, 94–106 and 130–142 in EgCaM. A phylogenetic tree showed the relationship of EgCaM with calmodulin from other parasites and hosts; EgCaM clustered with the calmodulins from *E. multilocularis* and *H. microstoma*, but not with the other calmodulins (Fig. 2).

### Expression, purification and western blotting analysis of EgCaM

**Fig. 3:**
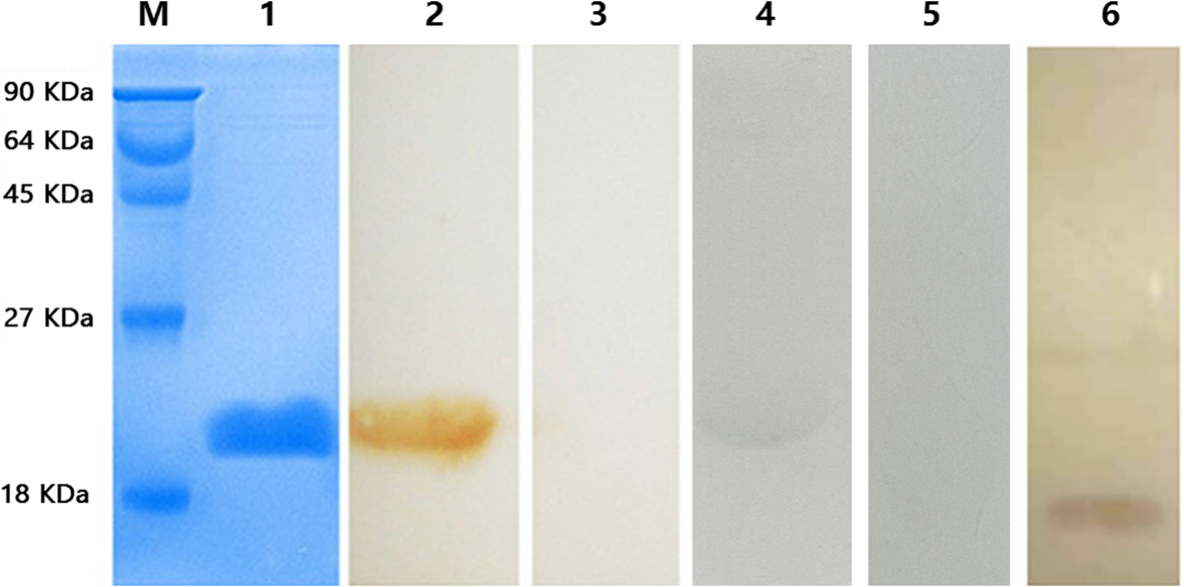
Purification and western blotting analysis of recombinant *E. granulosus* calmodulin (rEgCaM). Lane M: protein molecular weight markers; Lane 1: purified rEgCaM; Lane 2: rEgCaM was probed with *E. granulosus*-positive sheep sera; Lane 3: rEgCaM probed with sera of non-infected sheep; Lane 4: rEgCaM probed with anti-rEgCaM rabbit sera; Lane 5: rEgCaM probed with pre-immunized rabbit sera; Lane 6: total *E. granulosus* protoscoleces (PSCs) extract probed with rabbit anti-rEgCaM IgG

The pET28a(+) plasmid containing the EgCaM cDNA was confirmed by double digestion and DNA sequencing. Soluble EgCaM with a His-tag was expressed in *Escherichia coli* BL21 after 4 h of induction with 1 mM IPTG at 37 °C. The purified rEgCaM produced a single band of approximately 20 kDa (including the His-tag), which agreed with the predicted molecular weight (Fig. 3). In western blotting, rEgCaM could react with *E. granulosus* positive sheep sera and anti-rEgCaM rabbit sera. The specific band was visible, which was not observed following incubation of the membrane with sera of non-infected sheep or with pre-immunized rabbit sera (Fig. 3). In addition, total PSCs extract was blotted with rabbit anti-rEgCaM IgG and showed a protein of approximately 16.0 kDa. The size of this protein was similar to the theoretical molecular weight of EgCaM. It was concluded that EgCaM had a good antigenicity and immunoreactivity.

### Indirect ELISA

Building on the good antigenicity and immunoreactivity of EgCaM, the preliminary serodiagnostic potential of EgCaM based on indirect ELISA was assessed. The optimal concentration of the EgCaM antigen was 1.6 μg/well and the best dilution of the sera was 1:160. The cut-off value of the EgCaM-ELISA was 0.472 (mean = 0.354, SD = 0.040) which was inferred from the *E. granulosus-*negative sera of sheep. Based on the cut-off value, a total of 38 sheep sera (31 for *E. granulosus*-positive and seven for *T. multiceps*-positive) and 10 goat sera (*C. tenuicollis*-positive) were tested. Twenty-eight sera samples from sheep infected with *E. granulosus* were detected as positive, indicating a sensitivity of 90.3% (28/31). In the cross-reaction assay, the OD values of five *T. multiceps*-positive sera isolated from sheep and three *C. tenuicollis*-positive sera isolated from goat were lower than the cut-off value. This indicated that the specificity of this assay was 47.1% (8/17).

### Immunohistochemical localization of EgCaM in parasite sections

**Fig. 4:**
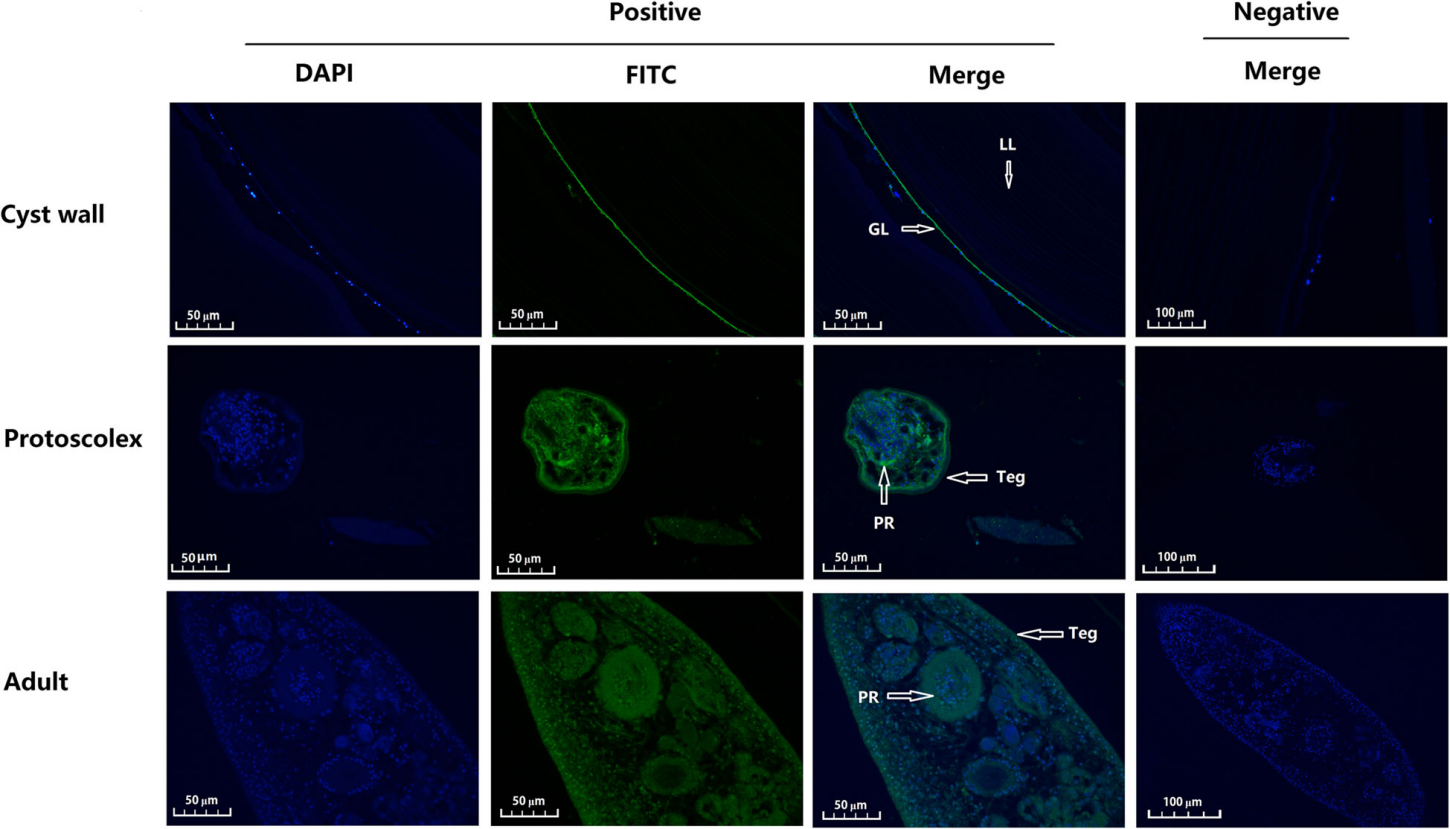
Immunohistochemical localization of *E. granulosus* calmodulin (EgCaM) in sections of *E. granulosus*. Anti-recombinant EgCaM (rEgCaM) rabbit IgG was used as the primary antibody and fluorescein isothiocyanate (FITC)-conjugated goat anti-rabbit IgG was used as the secondary antibody to detect the native protein in protoscoleces, the germinal layer, and adult worms. The nuclear DNA was stained with 2-(4-amidinophenyl)-1H–indole-6-carboxamidine (DAPI). Pre-immune rabbit sera was used as negative control. Images of protoscoleces are magnified at ×400; germinal layer and adult worm are magnified at ×200. *Abbreviations*: LL, laminated layer; GL, germinal layer; Teg, tegument; PR, parenchymal region

Anti-rEgCaM rabbit IgG was used to detect the native protein in protoscoleces, cyst walls and adult worms using immunofluorescence analysis (Fig. 4). Specific immunofluorescence was detected in almost all tissues, including the tegument and parenchymal region of the protoscoleces, the tegument and inner body of adult worms, as well as the germinal layer of cyst wall, suggesting that rEgCaM was ubiquitously expressed in all of the tissues of *E. granulosus* except for the laminated layer of the cyst wall. No specific fluorescence was observed in any sections when native rabbit IgG was used.

### mRNA expression of rEgCaM in PSCs treated with H_2_O_2_

The mRNA expression of rEgCaM in PSCs treated with 5 mM H_2_O_2_ was assessed by quantitative real-time PCR (qPCR) and normalized using the level of the *ACTB* mRNA.

**Fig. 5:**
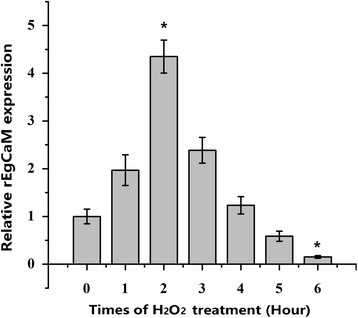
Relative expression levels of recombinant *E. granulosus* calmodulin mRNA (rEgCaM) in PSCs treated with H_2_O_2_ for different times. The mRNA expression of rEgCaM in PSCs treated with 5 mM H_2_O_2_ for 0, 1, 2, 3, 4, 5 and 6 h were assessed by qPCR analysis and normalized to the levels of the β-actin gene. Data are presented as the mean ± SD of triplicate experiments. Statistically significant differences between the 0 h group (as the control) and the other groups were determined using Student’s t-test (2 h: *t*_(7)_ = 3.832, *P* = 0.005; 6 h: *t*_(7)_ = -2.792, *P* = 0.000) (*P < 0.05*)

As shown in Fig. 5, the mRNA expression level of rEgCaM was increased at the start of H_2_O_2_ exposure and the level was upregulated by 4-fold after 2 h. The expression gradually decreased because of the increased apoptosis of the PSCs (Fig. 5).

### Biochemical characterization of rEgCaM

**Fig. 6:**
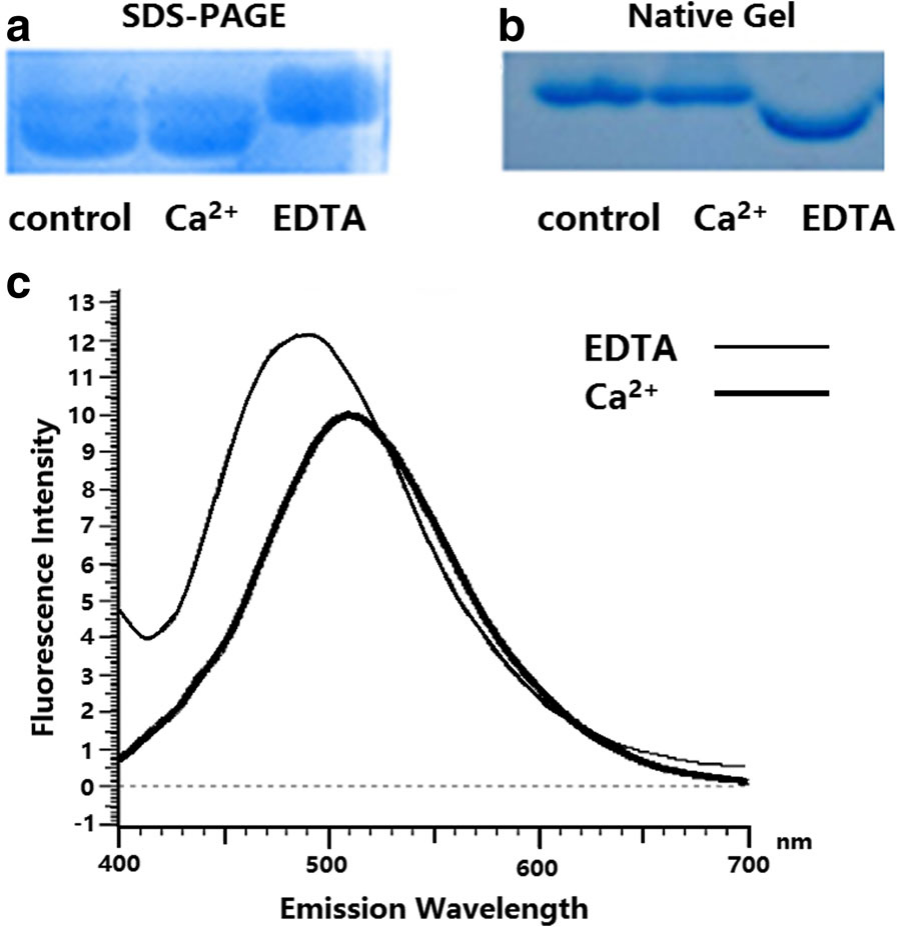
Analysis of Ca^2+^-binding properties of recombinant *E. granulosus* calmodulin (rEgCaM). The mobility of recombinant protein in the presence and absence of Ca^2+^ was compared on both SDS-PAGE (**a**) and native gel (**b**) electrophoresis. **c** 1-anilinonaphthalene-8-sulfonic acid (ANS) fluorescence emission spectra of rEgCaM

rEgCaM in the presence of Ca^2+^ demonstrated increased electrophoretic mobility on SDS-PAGE, compared with protein the treated with EDTA (Fig. 6a). On native polyacrylamide gels, the electrophoretic mobility of rEgCaM with Ca^2+^ was slower than that of protein exposed to EDTA (Fig. 6b). In SDS-PAGE and native gels, the control protein (untreated rEgCaM) showed the same mobility as rEgCaM in the presence of Ca^2+^, suggesting that the purified protein bound Ca^2+^ during the processes of expression or purification.

The maximum ANS fluorescence emission wavelength of rEgCaM in the presence of EDTA was 509 nm. The fluorescence intensity was enhanced when rEgCaM bound Ca^2+^ and the emission maximum moved to 490 nm (Fig. 6c), indicating that rEgCaM underwent conformational changes after binding Ca^2+^.

## Discussion

Calmodulin, as a dynamic Ca^2+^ sensor, is present in all eukaryotic cells and mediates a variety of cellular signaling activities, such as regulation of gene expression, enzymatic activities and mitochondrial events, modulation of ion channel activities, and specific mechanisms of synaptic transmission [16–18]. Although calmodulin has been widely studied and well characterized in many organisms, there are little data on the molecular and biochemical characterization of CaM in *E. granulosus* [19–23]. A number of calcium-binding “CaM-like” proteins have been identified in *E. granulosus*, but none of these has a “classical” CaM sequence or structure. CaM is typically a protein of 16 kDa, comprising two globular domains connected by a flexible alpha helix hinge [19]. The cDNA sequence encoding *E. granulosus* calmodulin was identified as encoding a “classical” CaM and uploaded to GenBank (accession code: KR153481). In the present study, we successfully expressed the “classical” CaM of *E. granulosus* in *E. coli*, characterized its locations in *E. granulosus* sections, assessed its mRNA expression of PSCs in different states, and assessed its serodiagnostic potential. Furthermore, we also determined the bioinformatic features and the ability of the protein to bind Ca^2+^ of rEgCaM.

Four Ca^2+^-binding domains in EgCaM at amino acid positions 21–32, 57–68, 94–106 and 130–142 demonstrated that this protein belongs to the EF-hand calcium-binding protein (CaBP) family, which contain two or more EF-hands domains [24]. Additionally, the amino acid sequence of EgCaM showed high identity with calmodulins from cestodes, trematodes, nematodes, protozoons and mammals (Fig. 2). Calmodulin was previously characterized as a highly conserved protein from the parasite *Schistosoma mansoni*; the alignment of both SmCaM1 and SmCaM2 shared 97–98% identity with other CaMs from mammals, flatworms and insects [19]. These data indicated that calmodulin has remained highly conserved across species during evolution. Moreover, mutations and the composition of the calmodulin intergenic spacer in *Leishmania* species have been studied as a molecular target that might have taxonomic value [25]. Moreover, Karabinos [26] found that CaM-like proteins of *C. elegans* arose from a CaM ancestor through repeated gene duplications, fusions and sequence divergence. In *E. granulosus*, CaM-like proteins showed a low identity with EgCaM; therefore, it would be meaningful to explore the relationships between CaM and CaM-like genes when more homologs of cestodes are included in the future.

Recently, increasing numbers of studies have used recombinant protein antigens in serological diagnosis methods to detect animals suspected of being infected [27–29]. It was important to evaluate the serodiagnostic potential of this protein to promote further research. In our study, western blotting analysis of EgCaM showed that this protein had a good antigenicity and immunoreactivity. However, even though sensitivity was high, the use of rEgCaM in the serodiagnostic ELISA lacked utility because of its low specificity. This might reflect the high conservation of CaM. In conclusion, the results revealed that rEgCaM was not a suitable as a diagnostic antigen.

Immunohistochemical localizations showed that rEgCaM was ubiquitously expressed in the larva, germinal layer and adult worm sections of *E. granulosus*. For example, EgCaM was expressed in the germinal layer, which is one of the most physiologically active regions of the cysts. It is possible that an unknown Ca^2+^-dependent mechanism occurs in this region. The distribution of EgCaM in the tegument of the adult stage indicated that the Ca^2+^ signaling pathway could function between *E. granulosus* and its host. Interestingly, a similar result was also reported in another flatworm *Clonorchis sinensis* [22]. Calcineurin, a Ca^2+^-calmodulin activated serine-threonine protein phosphatase, has been studied in protoscoleces and it was suggested that calcineurin is specifically involved in exocytic activity [30]. EgCaM was also expressed in the tegument tissues and parenchymal region of protoscoleces stage, implying that calmodulin is associated with exocytic activity in protoscoleces. These data indicated that EgCaM might play a crucial role in the growth and development of the parasite.

In other species, it has been proven that calcium ions and CaM are involved in abiotic stress responses including drought, salt stress, cold and heat stimuli [31–33]. However, there are few studies on the function of CaM in parasites dealing with abiotic stress. During development and proliferation in the host, *E. granulosus* must cope with oxidants and reactive oxygen species (ROS) derived from their own metabolism and from the host’s activated immune cells [34]. To further study the function of CaM in *E. granulosus* under the oxidative stress, PSCs were treated with 5 mM H_2_O_2_ for the indicated times up to 6 h at 37 °C. The mRNA expression level of rEgCaM was increased from the start of H_2_O_2_ exposure. The result suggested a role for EgCaM in the defense against oxidative stress. With increasing exposure time, the activity and metabolic rate of PSCs declined and the apoptosis or death of PSCs was enhanced. The mRNA expression of rEgCaM also decreased. Thus, it was demonstrated that CaM is related to the growth and metabolism of PSCs*.* The results suggested that calmodulin plays an indispensable role in many physiological functions in *E. granulosus*.

EgCaM is an EF-hand calcium binding protein with four Ca^2+^-binding domains, displaying changes in its electrophoretic mobility in the absence and presence of Ca^2+^. rEgCaM presented a typical Ca^2+^-induced electrophoretic mobility shift in this study (Fig. 6). Grab et al. [35] studied the effect of different divalent cations on migration through SDS-PAGE of purified canine and porcine brain calmodulins. Under denaturing condition, in the presence of Ca^2+^, the protein migrated much faster, which was consistent with our previous study [35]. He et al. [36] also came to the same conclusion when they invested CaM from *Sarcoptes scabiei*. In our study, rEgCaM in the presence of Ca^2+^ demonstrated increased electrophoretic mobility on SDS-PAGE, compared with the protein treated with EDTA (Fig. 6a). On native polyacrylamide gels, the electrophoretic mobility of rEgCaM with Ca^2+^ was slower than that of protein exposed to EDTA (Fig. 6b) which presented a typical Ca^2+^-induced electrophoretic mobility shift. These results were consistent with previous studies of CaBPs in *C. sinensis* and *Fasciola hepatica* [20, 22, 32]. Moreover, some calmodulins with mutations in their serine and tyrosine residues retain these characteristics [37]. One of the biochemical features of calmodulin is conformational change, exposing more hydrophobic residues on the protein surface after Ca^2+^ binding [38]. rEgCaM displayed this characteristic, which was verified using an ANS fluorescence assay. When Ca^2+^ was bound to rEgCaM, ANS fluorescence was enhanced, and the ANS fluorescence emission wavelength shifted from 509 nm to 490 nm.

## Conclusions

In this study, *E. granulosus* calmodulin was cloned, expressed and characterized. The antigenicity and immunoreactivity of rEgCaM were detected using western blotting and indirect ELISA. The results showed that this protein had a good antigenicity and immunoreactivity, but was not a suitable potential diagnostic antigen. rEgCaM was ubiquitously expressed in protoscoleces and adults of *E. granulosus*, as well as the germinal layer of cyst wall. The mRNA expression level of rEgCaM increased from the start of H_2_O_2_ exposure and then gradually decreased due to the increased apoptosis of PSCs. The Ca^2+^-binding properties of EgCaM were measured and rEgCaM presented a typical Ca^2+^-induced electrophoretic mobility shift. In conclusion, our study demonstrated that CaM might play an indispensable role in many physiological functions in *E. granulosus*.

## Abbreviations

ANS: 1-anilinonaphthalene-8-sulfonic acid
CE: Cystic echinococcosis
ELISA: Enzyme-linked immuno-sorbent assay
PBS: Phosphate-buffered saline
PSCs: *E. granulosus* protoscoleces
rEgCaM: Recombinant *E. granulosus* calmodulin
SDS-PAGE: Sodium dodecyl sulfate polyacrylamide gel electrophoresis
